# Specific post-translational histone modifications of neutrophil extracellular traps as immunogens and potential targets of lupus autoantibodies

**DOI:** 10.1186/ar3707

**Published:** 2012-02-02

**Authors:** Chih Long Liu, Stephanie Tangsombatvisit, Jacob M Rosenberg, Gil Mandelbaum, Emily C Gillespie, Or P Gozani, Ash A Alizadeh, Paul J Utz

**Affiliations:** 1 Department of Medicine, Division of Immunology and Rheumatology, Stanford School of Medicine, 269 Campus Drive, Stanford, California 94305, USA; 2 Center for Immunology, University of Minnesota Medical School, 420 Delaware Street SE, Minneapolis, Minnesota, 55455, USA; 3 Department of Medicine, Division of Oncology and of Hematology, Stanford School of Medicine, 300 Pasteur Drive, Stanford, California 94305, USA; 4 Department of Biology, Stanford University, 371 Serra Mall, Stanford, CA 94305, USA

## Abstract

**Introduction:**

Autoreactivity to histones is a pervasive feature of several human autoimmune disorders, including systemic lupus erythematosus (SLE). Specific post-translational modifications (PTMs) of histones within neutrophil extracellular traps (NETs) may potentially drive the process by which tolerance to these chromatin-associated proteins is broken. We hypothesized that NETs and their unique histone PTMs might be capable of inducing autoantibodies that target histones.

**Methods:**

We developed a novel and efficient method for the *in vitro *production, visualization, and broad profiling of histone-PTMs of human and murine NETs. We also immunized Balb/c mice with murine NETs and profiled their sera on autoantigen and histone peptide microarrays for evidence of autoantibody production to their immunogen.

**Results:**

We confirmed specificity toward acetyl-modified histone H2B as well as to other histone PTMs in sera from patients with SLE known to have autoreactivity against histones. We observed enrichment for distinctive histone marks of transcriptionally silent DNA during NETosis triggered by diverse stimuli. However, NETs derived from human and murine sources did not harbor many of the PTMs toward which autoreactivity was observed in patients with SLE or in MRL/*lpr *mice. Further, while murine NETs were weak autoantigens *in vivo*, there was only partial overlap in the immunoglobulin G (IgG) and IgM autoantibody profiles induced by vaccination of mice with NETs and those seen in patients with SLE.

**Conclusions:**

Isolated *in vivo *exposure to NETs is insufficient to break tolerance and may involve additional factors that have yet to be identified.

## Introduction

Neutrophil extracellular traps (NETs) were first described in 2004 as web-like structures that trap and neutralize microbes at sites of infection [1]. Neutrophils, a first line of defense against microorganisms during such encounters, produce these highly modified chromatin webs through a cellular suicide program distinct from apoptosis and necrosis, termed "NETosis" [2]. In addition to neutrophil antimicrobial proteins, NETs are comprised of chromatin components, including histones. Because NETs are extracellular and typically in an inflammatory environment, their proximity to components of the adaptive and innate immune systems might provide an immunogenic substrate for autoimmune responses during regular encounters with commensal and pathogenic microbes.

Indeed, an emerging and growing body of literature supports a putative link between NETs and autoimmunity. Baker *et al*. identified circulating NETs in the blood of pediatric patients with malaria, a subset of whom also exhibited signs of an autoimmune response [3]. A more recent study identified a subset of patients with lupus nephritis whose sera were impaired in degrading NETs, suggesting that such impairment could be pathogenic [4]. Two recent studies reported activation of plasmacytoid dendritic cells (pDCs) by complexes between NETs and antimicrobial peptides such as LL-37 that engage Toll-like receptor 9 (TLR9) and result in Type I interferon production, a process known to be associated with SLE [5,6].

Anti-histone antibodies are found in 70% to 80% of patients with idiopathic SLE [7], and in more than 90% of patients with drug-induced lupus [7,8]. Furthermore, the presence of such antibodies is a highly specific serological feature that distinguishes both of these lupus variants from other autoimmune diseases [9,10]. Patients with drug-induced lupus due to procainamide or hydralazine most commonly do not have antibodies directed against non-histone nuclear antigens, a serological feature frequently used to distinguish between drug-induced and idiopathic SLE [7,11-13]. Further, the tendency and degree to which such drugs are covalently modified by acetylation (a common post-translational modification of histones) critically influences their tendency to induce anti-histone antibodies and lupus [14,15]. Finally, the capacity of several drugs to serve as neutrophil myeloperoxidase substrates *in vitro *is associated with their ability to induce lupus *in vivo *[16]. Collectively, these findings suggest potential mechanisms linking post-translational histone modifications to induction of autoimmunity, with neutrophils contributing to this process.

Over the last decade, a significant body of emerging evidence has supported a role for PTMs of several autoantigens in the pathogenesis of diverse autoimmune diseases [17,18]. Modified autoantigens have been shown to relocalize to other cellular compartments including apoptotic blebs during cell stress [19]. Such modified autoantigens have been proposed to elicit immune responses because they appear foreign to T and B cells or because the modifications may alter their processing and presentation by antigen presenting cells (reviewed in [20]). For example, many diverse autoantigens are substrates for cleavage by caspases, and some autoantibodies are better able to recognize cleaved antigens than native counterparts [21]. Similarly, we and others have shown that many different antigens are phosphorylated: for instance, transient phosphorylation of serine/arginine-rich (SR) splicing family members during apoptosis leads to their association with the U1-snRNP and U3-snoRNP autoantigen complexes, and can commonly be recognized by SLE sera in a phosphorylation-dependent manner [22-27].

However, to date, few studies have specifically examined the role of post-translational modifications (PTMs) in the context of NETs within SLE. For instance, while van Bavel and colleagues identified in a subset of patients with SLE autoantibodies against acetylated histone H2B tails [28], histone H4 [29] and histone H3K27Me3 [30], the relationship of these marks to those within NETs remains unclear, and SLE autoantibodies may recognize other histone PTMs. Such PTMs may play an important role in SLE pathogenesis, since a unifying characteristic of most SLE-associated autoantigens is that they contain either DNA or RNA with many of the associated protein components targeted by PTMs [17].

While NETs represent a strong candidate as a source of diverse exposed cryptic epitopes that may lead to autoimmunity, only a single PTM found on NET histones has been well-characterized. Specifically, NET histones harbor citrulline residues, a PTM mediated by the peptidyl arginine deiminase (PAD) family of enzymes during reversible deimination of arginine residues [31,32]. Autoantibodies directed against citrullinated proteins are highly specific at diagnosis of rheumatoid arthritis (RA), and have also been found in a collagen-induced arthritis model of RA [26,27]. Citrullination of histones arising from PAD-4 activity during NETosis was recently shown to be a specific marker of NETs and necessary for NET formation [31,32]. Accordingly, antibacterial innate immunity is considerably inhibited in *PAD-4*-deficient mice [33]. To date, while the protein components of NETs have been systematically identified [34], no studies have broadly profiled the PTM state of their histones.

We, therefore, hypothesized that NETs and unique associated histone PTMs are capable of inducing autoantibodies that target histones and lead to subsequent autoimmunity. We devised novel and efficient methods for production, characterization and visualization of NETs *in vitro*. We biochemically characterized the PTMs accompanying *in vitro *NETosis and broadly profiled the *in vivo *humoral immune responses of patients with SLE and mice immunized with NETS by applying multiple proteomic approaches, including autoantigen microarrays [35], PTM-modified histone peptide arrays [36] and a high-throughput immunoblotting assay (modified Multiple Antigen Blot Assay, MABA) [37]. Consistent with recent findings [28], we found that sera from patients with SLE reacted to acetyl-H2B histone proteins. In broadly profiling the PTMs of NETs from human and mouse sources, we observed their enrichment for distinctive PTMs characteristic of transcriptional silencing. However, these marks only partly overlapped with autoantibody profiles in histone-reactive sera of patients with SLE and with those in sera from mice prone to spontaneous autoimmunity. Nonetheless, we found that NETs could serve as weak autoantigens *in vivo*, capable of eliciting mouse IgG and IgM responses.

## Materials and methods

### Human subjects, specimens and controls

In accordance with approved Institutional Review Board protocols, serum samples from patients with SLE were obtained with informed consent from the Autoimmune Biomarkers Collaborative Network (ABCoN), a multi-disciplinary, multi-institutional effort to identify clinically useful biomarkers for the management of autoimmune diseases [38-40]. Normal sera and neutrophils were similarly obtained from healthy donors as part of the Stanford Chronic Immunologic Disease Registry and Repository and IRB protocol #17036, respectively. Human neutrophils were isolated from peripheral blood as previously described [34], using Percoll (GE Healthcare) density gradient separation. A mixture of commercially available autoimmune sera with defined reactivities was used as a positive control (ImmunoVision, Springdale, AR), and secondary antibody alone was used as a negative control.

### Mice and NET immunization protocol

All animal experiments were approved by, and performed in compliance with, a protocol approved by the Institutional Animal Care and Use Committee of Stanford University. Female Balb/c mice 9 to 12 weeks of age were obtained from the Jackson Laboratory (Bar Harbor, ME). All mice used in this study were maintained under standard conditions at the Stanford University Research Animal Facility. NETs from the EPRO cell line (derived by differentiation from EML cells) were prepared as described below. NETs were prepared and stored at a concentration of 10 μg/ml of which 200 μl was subcutaneously injected at each immunization. Each treatment group included 3 mice, immunized weekly over 4 weeks with NETs alone, or NETs combined with murine cathelicidin-related antimicrobial peptide (CRAMP) at a 5:1 w/w ratio to NETs [41]. Proteinuria was assessed by dipstick analysis using Albustix (Bayer, Pittsburgh, PA). Mouse serum samples were obtained by saphenous vein bleeding immediately before the first injection and once every 4 weeks after immunization for up to 12 weeks.

### Cell culture and differentiation of neutrophils

Maintenance and differentiation of myeloid cell lines into neutrophils were performed at 37°C and 5% carbon dioxide as previously described [42]. Briefly, the human promyelocyte HL-60 cell line was obtained from ATCC (Manassas, VA; #CCL-240) and maintained in RPMI 1640 (Invitrogen, Carlsbad, CA) media supplemented with 10% fetal bovine serum (FBS), 2 mM L-glutamine, 25 mM hydroxyethyl piperazineethanesulfonic acid (HEPES) and 1X penicillin/streptomycin (P/S). Cells were maintained at a density range of 1 × 10^5 ^to 8 × 10^5 ^cells/ml for a maximum of 80 passages, and differentiated for 3 days into neutrophils from a starting density of 3 × 10^5 ^cells/ml with a final concentration of 1 μM all-trans retinoic acid (ATRA; Sigma, St. Louis, MO) + 1.25% dimethylsulfoxide (DMSO).

The murine multipotent cell line EML (Clone 1) was obtained from ATCC (#CRL-11691) and maintained in Iscoveís modified dulbeccoís medium (IMDM) media (Invitrogen) supplemented with 20% horse serum (Omega Scientific, Tarzana, CA), 2 mM L-glutamine, 1X P/S, and 15% stem cell factor (SCF) containing conditioned media (from BHK/MKL cell supernatants, detailed below). EML cells were maintained in 6-well plates at a density range of 1 × 10^5 ^to 5 × 10^5 ^cells/ml and differentiated for two days by adding a final concentration of 10 μM ATRA and 25 ng/ml recombinant murine IL-3 (Peprotech, Rocky Hill, NJ). The cells were further differentiated for one day with fresh media containing a final concentration of 60 μM ATRA and 150 ng/ml recombinant murine IL-3. After washing the cells twice with PBS, they were grown in IMDM media (Invitrogen) supplemented with 20% horse serum (Omega Scientific), 1X P/S and 10% BHK/HM-5 conditioned medium as a source of GM-CSF, for 9 days without splitting cells. At this stage, EML cells had differentiated into EPRO cells, which were maintained in this medium at a density of 0.5 × 10^5 ^to 8 × 10^5 ^cells/ml. EPRO cells were differentiated for 3 days into neutrophils from a starting density of 3 × 10^5 ^cells/ml with a final concentration of 10 μM ATRA.

The murine promyelocyte cell line MPRO [43] was obtained from Dr. Tsai (University of Utah) and maintained at a density range of 0.5 × 10^5 ^to 1.0 × 10^6 ^cells in IMDM media supplemented with 20% horse serum, 1X P/S, and 10% BHK/HM-5 conditioned medium as a source of GM-CSF, and differentiated for 3 days into neutrophils from a starting density of 3 × 10^5 ^cells/ml with a final concentration of 10 μM ATRA.

### Conditioned media

Growth factors required for EPRO (EML-derived) and MPRO cultures were obtained using two secreting cell lines (generous gifts from Dr. Tsai, University of Utah) and prepared as previously described [42]. Briefly, baby hamster kidney HM-5 (BHK/HM-5) cells, which secrete murine GM-CSF, or baby hamster kidney MKL (BHK/MKL) cells, which secrete murine SCF, were maintained in DMEM high glucose media (Invitrogen) supplemented with 10% heat-inactivated FBS off US origin (Omega Scientific), 2 mM L-glutamine, and 100 U/ml penicillin/100 μg/ml streptomycin (1X P/S). These were expanded to T-175 flasks and grown to confluence. Cell culture supernatants were harvested when the media turned yellow-orange, then sterile-filtered and frozen at -20°C until ready for use.

### Stimulation and isolation of NETs

Neutrophils derived from cell lines or isolated from human donors were stimulated to produce NETs as previously described [2,34]. Neutrophils were incubated at 37°C with 5% carbon dioxide, in 100 mm plates (up to 10 ml volume) or 150 mm plates (10 to 40 ml volume) at 1.5 × 10^6 ^cells/ml in serum-free RPMI media lacking phenol red (Invitrogen) and stimulated with one of the following stimulants at final concentration: 30 nM phorbol myristate acetate (PMA), 8 μM ionomycin, 10 mM hydrogen peroxide, 7.0 ng/ml TNF (7.4 ng/ml for EPRO), or 250 ng/ml lipopolysaccharide (LPS). At 2 hours (additionally, at 0 hours for primary human neutrophils), protease inhibitor cocktail (Sigma, #P1860) was add at 1:200 dilution. After 2 to 3 more hours, cells and NETs were detached with a cell scraper, transferred to 15 ml or 50 ml conical tubes, and pelleted by spinning at 1000 × *g *for 5 minutes. To gently digest the NETs, the supernatant was discarded and the pellet resuspended in micrococcal nuclease (MNase; Worthington Biochemical, Lakewood, NJ) digestion mixture containing 5 U/ml MNase and 10 mM calcium chloride in 1X PBS (and additionally, protease inhibitor cocktail for primary human neutrophils). This mixture was gently pipetted until the pellet fully dissolved, typically in 30 seconds. The mixture was then further incubated at 37°C in a heat block for 30 seconds, and pelleted by centrifugation at 3000 × g for 1 minute. The cleared supernatant was used as the chromatin fraction in subsequent analyses.

### Quantitation of NETs and apoptosis assays

An aliquot of NETs and of unstimulated neutrophils (about 2 to 3 × 10^6 ^cell equivalents each) were separately processed in parallel with the Qiagen DNeasy Blood and Tissue kit (, Valencia, CA) following the manufacturer's instructions to obtain genomic DNA. NET yield was determined with a Nanodrop 1,000 UV spectrophotometer (NanoDrop, Wilmington, DE) and calculated from the ratio of DNA obtained from NETs relative to that obtained from the corresponding unstimulated neutrophils. NET DNA prepared in this fashion was also separated by electrophoresis on a 2% tris-acetate-EDTA agarose gel with 0.3 μg/ml ethidium bromide to ascertain the degree of NET digestion. Apoptosis accompanying NETosis was measured by Caspase-Glo chemiluminescent assay (Promega, Fitchburg WI) during a 4-hour NET stimulation assay induced by hydrogen peroxide within primary human neutrophils or HL-60 derived neutrophils. Staurosporine was used as a positive apoptosis control and was also compared with untreated neutrophils resting over the same interval.

### Fluorescence imaging of NETs

Indirect fluorescence of NETs using 4',6-diamidino-2-phenylindole (DAPI) stain was performed as described in [44], using FBS heat-inactivated at 70°C for 30 minutes to avoid bovine serum nucleases from degrading NETs [45]. Images were acquired using a Leica DM5000 microscope with a HCX PL Fluotar 40X/0.75 oil objective, using a QImaging Scientific Retiga EXi Fast 1394 digital capture camera with RGB Slider, with the QCapture Pro Version 5.0 image capture software. Background color inversion was performed using Adobe Photoshop Version 7.0.

### Immunoblot profiling of NET post-translational modifications

NETs were analyzed with a panel of 41 antibodies specific to both unmodified histones and histones modified with various post-translational modifications (Additional file 1: Supplemental Table 1), using a MABA based on the Miniblotter apparatus (Immunetics, Cambridge, MA) [37,46]. Unstimulated neutrophils were lysed in RIPA buffer (150 mM sodium chloride, 1.0% NP-40, 0.5% sodium deoxycholate, 0.1% sodium dodecyl sulfate (SDS), and 50 mM Tris-HCl, pH 8.0; supplemented with a protease inhibitor Complete tablet (1 mini tablet per 10 ml; Roche, Basel, Switzerland) and sonicated for 20 seconds at 50% duty cycle (one second on, one second off) and 13% amplitude with a digital sonifier (Branson, Danbury, CT).

Sonicated cell lysates and digested NETs were each combined with sample loading buffer (final concentration 2% SDS, 50 mM Tris-HCl pH 6.8, 6% glycerol, 1% beta-mercaptoethanol and 0.008% bromophenol blue), denatured at 100°C for 5 minutes, then separated in an 18% SDS-PAGE gel using a 2-D preparative gel comb (one gel-width lane plus one small lane for protein marker). The gel was transferred onto a polyvinylidene fluoride (PVDF) membrane (Millipore, Billerica, MA) using a semi-dry transfer system, blocked with blotting-grade blocker (Bio-Rad, Hercules, CA), washed in 1X tris-buffered saline plus 0.1% tween (TBST).

The blocked and washed membrane was assembled in a 28-lane Miniblotter apparatus following the manufacturer's instructions. Up to 22 antibodies at the appropriate dilution (Table S1) from the panel were applied, one in each lane, with empty lanes applied with 1X TBST. The apparatus was then sealed and the Miniblotter was incubated at 4°C overnight on a rocking platform. The Miniblotter was washed with 60 ml 1X TBST using its vacuum manifold. The membrane was removed and washed once for 15 minutes in TBST, then 3 times for 5 minutes in TBST. A mixture of horse radish peroxidase (HRP)-conjugated donkey anti-rabbit secondary antibody and HRP-conjugated goat anti-mouse antibody (Jackson Immunoresearch, West Grove, PA), each at 1:25,000 dilution in TBST, was applied to the membrane and incubated for 1 hour. Following washes in TBST (15 minutes once, then 5 minutes for 3 times), enhanced chemiluminescent (ECL) detection reagent (GE Healthcare) was applied, and the blot was developed on film.

### Microarray autoantibody profiling

Detailed autoantibody profiling protocols and a list of arrayed antigens have been published previously [35]. Briefly, autoantigens were printed in ordered arrays on nitrocellulose-coated FAST slides (Whatman, Piscataway, NJ) at 0.2 mg/ml concentration using a Versarray ChipWriter Pro Robotic Arrayer (Bio-Rad). Individual arrays were blocked with 1% blocking-grade blocker (Bio-Rad) in PBS for 1.25 hours on a rocking platform at room temperature. Arrays were probed with 400 μl human or mouse serum diluted 1:250 in 1X PBST with 5% FBS for 1.5 hours on a rocking platform at 4°C, followed by washing and incubation with a 1:2000 dilution of DyLight 649-conjugated goat anti-human or goat anti-mouse IgG secondary antibody (Jackson Immunoresearch). Arrays were scanned using a GenePix 4000B scanner, always using the same PMT power for a given set of arrays. The net mean pixel intensities of each feature were determined using GenePix Pro 6.1 software (Molecular Devices, Sunnyvale, CA). Epigenome peptide microarrays were probed with mouse serum as described [36]. All microarray data have been deposited in the Gene Expression Omnibus (GEO) as GSE32544.

### Statistical methods

Data were expressed as mean net fluorescence intensity (MFI) units, representing the mean values from six replicate antigen or peptide features on each array. Histone-reactive SLE samples were defined as having a minimum normalized IgG MFI of 10,000 in at least one whole-protein histone antigen and histone-nonreactive samples were defined as having a maximum normalized IgG MFI less than 1,000 in any whole-protein histone antigen. Significance Analysis of Microarrays (SAM) [47] was applied to the dataset (with the MFI value of undetected array features set to 1) using the Wilcoxon signed-rank test statistic to identify antigens with statistically significant differences in array reactivity between different groups of human patients at False Discovery Rate of 0. Datasets were hierarchically clustered using Cluster 3.0 [48], using Euclidean distance and average linkage, with the MFI value of undetected array features set to 0 and with positive control samples excluded from being weighted in the clustering process. Results were displayed using Java TreeView software [49]. Significance of overlap between mouse IgM and IgG autoantigen reactivity was determined using cumulative hypergeometric distribution with a total population size of 122 antigens and an overlap of 28 to 36. The Euclidean distance between IgM and IgG profiles was calculated from their MFI values, with nominal *P*-value determined using a paired two-tailed t test.

## Results

### Distinctive profiles of SLE sera with and without reactivity toward histones

To test the hypothesis that NETs and the histone PTMs that they harbor might be capable of inducing anti-histone autoantibodies, we first tested serum samples from the ABCoN, a well-studied cohort of adult patients with SLE [38-40]. We randomly selected serum samples of 20 patients from a larger cohort profiled using autoantigen microarrays (data not shown), comprising 14 histone-reactive samples and 6 histone-nonreactive samples. Using the Human Epigenome Microarray Platform (HEMP) [36], we compared these SLE sera to each other as well as to control sera from nine healthy adults and to a positive control comprising a mixture of autoimmune sera with defined reactivity.

**Figure 1:**
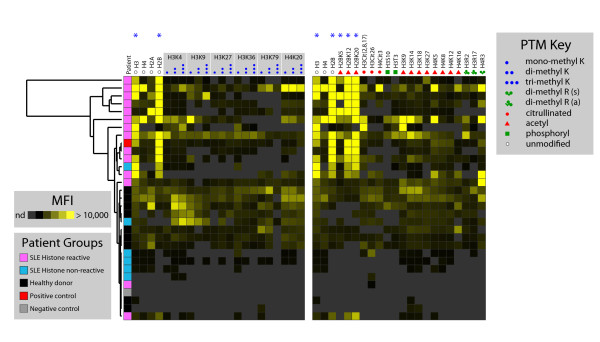
**Histone-reactive SLE IgG autoantibodies recognize acetyl-H2B**. Sera from patients with SLE or healthy subjects were profiled on Human Epigenome Microarray Platform (HEMP) and probed with a secondary anti-human IgG antibody. Samples (rows) are clustered hierarchically based on corresponding reactivity profiles to the depicted peptide epitopes from HEMP arrays (columns). The depicted histone peptides (columns) were selected to match those interrogated by immunoblot assays in Figure 4 and Additional file 1: Supplemental Figures 2 and 4, with specific PTMs epitopes shown at top according to the PTM key. Heatmap tiles reflect magnitude of IgG autoantibody binding reactivity, according to the mean fluorescence intensity (MFI) intensity scale as indicated. The left heatmap panel consists primarily of peptides containing mono-, di- and tri-methyl lysine PTMs, while the right panel consists of peptides containing a variety of PTMs, including citrulline, acetyl, phosphoryl, and both symmetric (*s*) and asymmetric (*a*) di-methyl arginine. Blue asterisks (*) mark significant differences in autoantibody binding reactivity between histone positive samples and a combined group of histone negative and healthy control samples, as determined by Significance Analysis of Microarrays (SAM). IgG, immunoglobulin G; K, lysine; MFI, mean fluorescence intensity; nd, not detected; PTM, post-translational modification; R, arginine; SLE, systemic lupus erythematosus.

Using SAM, we identified IgG reactivity to nine peptides that significantly distinguish histone-reactive from -nonreactive sera among 96 peptides profiled (False Discovery Rate = 0). These reactivities included 5 of 41 peptides for which specific, commercially-available antibodies were tested (Figure 1), as well as 4 others (that is, H2BK15Ac, H3K18Me3K36Me3, H2BK5Me1, and H2BK5Me2). Hierarchical clustering of serum samples resulted in grouping of most of the histone-reactive sera, along with the positive control, with the clustering driven by reactivity to unmodified histone H2B peptide as well as H2B peptides acetylated at K12 and K20. Collectively, these findings are consistent with a previously published study reporting SLE autoantibody reactivity to apoptosis-related acetyl-H2B epitopes [28].

IgM autoantibody profiles exhibited similar reactivity patterns in the mixed PTM peptide panel (Additional file 1: Supplemental Figure 1, right panel), including acetyl peptides of histones H2B, H3 and H4, again consistent with prior studies [28,29]. We also observed considerable reactivity to multiple methyl-histone H3 peptides (Additional file 1: Supplemental Figure 1, left panel). However, both histone reactive and healthy sera shared this reactivity pattern and thus were not determined to be significant by SAM. Given that IgM antibodies tend to be lower affinity with broader cross-reactivity and may occur naturally with potential regulatory and protective roles against autoimmunity [50], the significance of reactivity among these epitopes is less clear. Modest levels of IgG or IgM reactivity to citrullinated H3 and H4 peptides were also observed, with no significant difference between SLE and healthy sample groups.

### Efficient production and visualization of NETs *in vitro*

**Figure 2:**
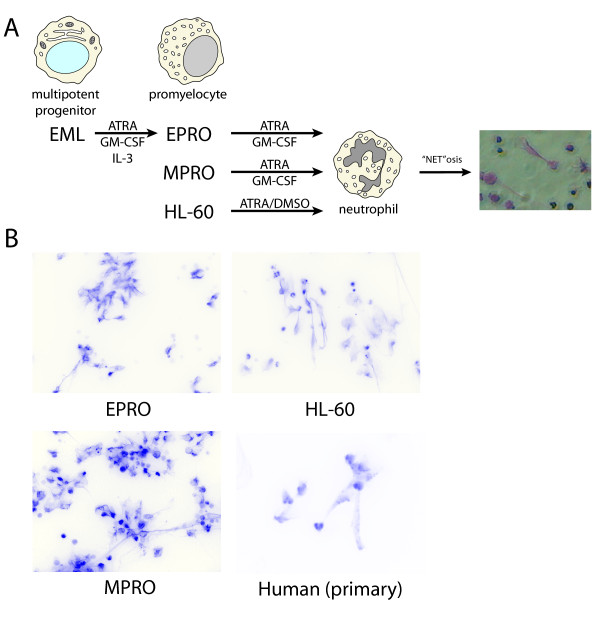
**Generation and visualization of neutrophil extracellular traps (NETs) from myeloid cell lines and human primary neutrophils**. **A**. Three cell line models used to generate neutrophils and NETs. Murine cell lines EPRO and MPRO were induced with ATRA and GM-CSF to differentiate into neutrophils. EPRO cells were generated by differentiating the EML multipotent progenitor cell line with ATRA, GM-CSF and IL-3. NETs were generated by stimulating cell line-derived or primary human neutrophils with hydrogen peroxide. **B**. Fluorescent (DAPI) images of NETs produced from neutrophils derived from EPRO, HL-60 or MPRO cell lines, or from primary human neutrophils. Color channels are inverted to yield white background for improved contrast. 400× magnification. ATRA, all-trans retinoic acid; DAPI, 4',6-diamidino-2-phenylindole; DMSO, dimethyl sulfoxide; GM-CSF, granulocyte macrophage colony-stimulating factor; IL-3, interleukin-3.

We next devised methods for generating NETs from human and murine myeloid cell lines, to facilitate the broad and uniform characterization of PTMs on human and murine NETs and to provide a supply of NETs for testing their immunogenicity *in vivo *(Figure 2). We employed two sources of murine and two sources of human NETs, including two murine cell lines derived from cells arrested in early myeloid differentiation (MPRO/promyelocyte; EML/multipotent progenitor), the human promyelocytic leukemia cell line HL60 as well as mature human granulocytes from healthy adult donors. To culture and differentiate the three immature cell lines into neutrophils, we adapted previous methods [34,42,44] then stimulated them as well as primary human neutrophils to produce NETs using hydrogen peroxide, TNF and LPS. We then visualized the resulting NETs using fluorescence microscopy (Figure 2). The corresponding NET DNA was characterized by gel electrophoresis (Additional file 1: Supplemental Figure 2) and the NET DNA yield from these diverse sources was quantified (Additional file 1: Supplemental Table 1). The DNA yield of purified NETs relative to unstimulated neutrophils varied depending on the preparation source and type of stimulus used for NETosis, ranging from < 10% for MPRO, 15% to 50% for EPRO (differentiated from EML cells), 9% to 45% for HL-60 and > 70% for primary human neutrophils. Importantly, we found that induction of NETosis in HL-60 cells and primary human neutrophils did not result in a significant degree of apoptosis (Additional file 1: Supplemental Figure 3).

### Post-translational modification profiles of human and murine NETs

To assess the immunogenicity of NETs in the context of autoantibody binding reactivity profiles observed (Figure 1), we biochemically characterized the PTMs present on NETs by employing a high-throughput immunoblotting assay (modified MABA [37]) that allows such profiling in a parallel manner using a MiniBlotter apparatus during primary antibody incubation. A set of 22 commercially available PTM-specific anti-histone antibodies was used to probe each immunoblot membrane, with a total of 44 epitopes (41 unique; Additional file 1: Supplemental Table 2) profiled in two separate panels. The two panels comprised one for profiling histone tail methylation (Additional file 1: Supplemental Figure 4A) and a second for capturing a variety of other histone PTMs (Additional file 1: Supplemental Figure 4B). We note that the analysis of NET PTMs is somewhat dependent on the conditions of each experiment, since the production of NETs coincides with neutrophil protease release.

**Figure 3:**
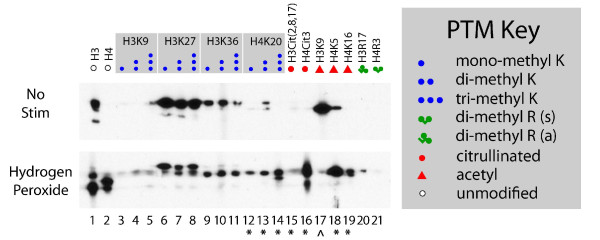
**Post-translational histone modifications of neutrophil extracellular traps (NETs) derived from primary human neutrophils**. Primary human neutrophils were isolated from peripheral blood, immediately stimulated with 10 mM hydrogen peroxide for 4 hours, and NETs were harvested. NETs or the corresponding unstimulated neutrophils were assayed by MABA. A panel of anti-histone antibody epitopes (top) is shown, with PTMs according to the PTM key. A more limited PTM panel was selected on the basis of available epitopes not subject to proteolysis by considering those enriched or depleted during NETosis of neutrophils derived from human and murine cell lines (Figure 4 and Additional file 1: Supplemental Figure 4). Bottom: asterisks or carats indicate increase or decrease (respectively) of band intensity for indicated lane in NETs compared to unstimulated neutrophils. Band migration is shown for 10-15 kDa range and film exposure time was 20'. MABA, Multiple Antigen Blot Assay; PTM, post translational modification.

We next compared chromatin preparations from primary human neutrophils and unstimulated HL-60 derived neutrophils to NETs derived from these same neutrophils following stimulation with hydrogen peroxide (Figure 3 and Additional file 1: Supplemental Figure 4). HL-60 derived neutrophils were also separately stimulated with TNF or LPS to induce NETs (Additional file 1: Supplemental Figure 4). During NETosis of primary human PMNs we observed significant proteolysis of the core histone proteins, limiting the availability of many histone PTMs (data not shown), as has been previously reported [34]. Therefore, for primary human PMNs, we used a more limited PTM panel selected on the basis of epitopes whose states were observed to be different in NETs compared to unstimulated neutrophils derived from cell lines and not subject to degradation. NETs derived from primary human neutrophils were enriched for several histone PTM methylation, citrullination and acetylation marks, including H4K20Me1/2/3, H3Cit(2,18,17), H4Cit3, H4K5Ac, and H4K16Ac, while depleted for H3K9Ac.

For comparison, HL-60 NETs were depleted of several PTMs associated with active transcription including H3K27Ac, H3K36Me2, H2BK12Ac, and H3K9Ac (Additional file 1: Supplemental Figure 4), and conversely, enriched for PTM marks associated with transcriptional inactivation during NETosis, including mono-, di- and tri-methyl H3K27. Further, levels of di-methyl arginine modification also decreased to nearly undetectable levels in HL-60 derived NETs, consistent with deimination of the arginine to citrulline during NETosis. Unexpectedly, however, we observed a moderate decrease in histone H3 citrullination during NETosis of HL-60 derived neutrophils, distinguishing them from primary human PMNs which induced a strong citrullination signature upon NETosis. Since we observed hypercitrullination in NETs derived from primary human neutrophils, our HL-60 population could conceivably harbor genotypic or phenotypic differences from HL-60 cells characterized in previous reports [31,32], accounting for the discordance with previously published observations.

In comparing the PTMs found in human NETs to epitopes recognized by serum autoantibodies from patients with SLE, many but not all autoantibody-reactive PTMs of SLE were also found in NETs. For example, while acetyl-H3 at K14 and K18 were recognized by serum IgG autoantibodies and also detected in NETs, acetyl-H3K9 and dimethyl-H4R3 (symmetric) were recognized but were only weakly positive or not detected in NETs derived from HL-60 cells. Many PTMs present in NETs (for example, other acetyl-H3; acetyl-H4) only exhibited modest IgG serum reactivity, which was present in both healthy controls and SLE patients. Serum IgM reactivity (Additional file 1: Supplemental Figure 1, left panel) was more widespread and thus overlapped to a greater extent with PTMs detected in NETs (many methyl-H3 PTMs, Additional file 1: Supplemental Figure 4). However, we observed this pattern both in patients with SLE known to have IgG histone auto-reactivity and healthy subjects, indicating that these observed IgM reactivities may not be indicative of a disease state.

**Figure 4:**
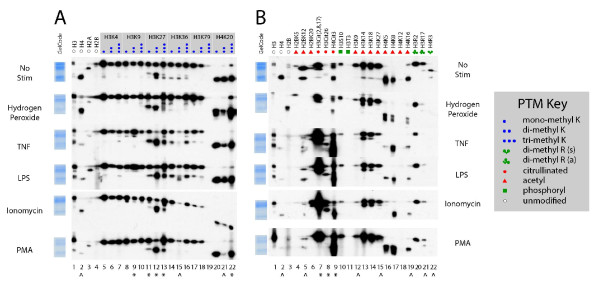
**EPRO-derived neutrophil extracellular traps (NETs) contain histone post translational modification (PTM) marks consistent with transcriptional silencing and hypercitrullination**. Murine cell-line derived neutrophils were stimulated and analyzed as in Figure 3 with additional stimulants including ionomycin and PMA. A panel of anti-histone antibody epitopes (top) matching those in Additional file 1: Supplemental Figure 4 is shown, with PTMs according to the PTM key. A strong and saturated band is notable with H3Cit(2,8,17) in EPRO NETs. Film exposure time for all cells were as described in Figure 3 (additional stimulants: 2' for ionomycin and 90' for PMA). PMA, phorbol myristate acetate.

Next, we biochemically characterized the PTMs present on EPRO-derived murine NETs with a goal of testing their immunogenicity *in vivo*. EPRO-derived NETs were prepared as for human cells described earlier and subjected to ionomycin and phorbol myristate acetate (PMA) as two additional stimuli (Figure 4). When comparing HL-60 and EPRO-derived NETs, the majority of the histone PTMs were consistent during NETosis and also similar in response to diverse stimuli. Specifically, in comparing EPRO-derived NETs to corresponding unstimulated neutrophils, we observed an even more striking pattern of PTMs associated with a transcriptionally silent state than for HL-60 cells. Furthermore, we observed an increase in the silencing marks di-methyl H3K9 and tri-methyl H4K20. In contrast with HL-60 derived NETs, however, we observed a strong citrullination signature (Figure 4B) in NETs prepared from EPRO-derived neutrophils, clearly demonstrating abundant citrullination of histones H3 and H4 and consistent with previous studies and primary human PMNs.

### Murine NETs are weakly immunogenic in vivo

We next tested the immunogenicity of NETs derived from the murine EPRO cell line *in vivo*. NETs prepared using hydrogen peroxide stimulation of EPRO cells were subcutaneously injected into two groups of female BALB/c wild-type mice weekly over 28 days, with one group receiving NETs alone, and another receiving NETs in combination with CRAMP, the murine analogue of LL-37 [41]. Trace or low levels of urinary protein were measured during the remainder of the time course, suggesting a weak response to the NET immunization (Additional file 1: Supplemental Table 3). Autoantibody profiles reflected reproducible and time-dependent responses for IgG and IgM and targeted diverse auto-antigens (*n *= 36 and *n *= 38, respectively; Additional file 1: Supplemental Figure 5A-C), although these responses were modest and transient. Reactivity to 28 antigens was observed for both IgG and IgM isotypes (*P *= 1.88 × 10^-12^), including many components within neutrophils or NETs, such as myeloperoxidase, catalase, histones (including H1 and H3), as well as single and double-stranded DNA. Nonetheless, mice immunized with NETs showed significant differential IgM and IgG reactivity towards many of the same antigens, as well as others (Additional file 1: Supplemental Figure 5D). Furthermore, we observed reproducible IgG seroreactivity targeting human IgG in mice immunized with NETs derived in serum free conditions, possibly reflecting a species cross-reactive rheumatoid factor activity precipitated by citrullinated murine NETs. Taken together, the autoantibody reactivity profiles are consistent with the antigenic components of the NET immunogen but suggest a modest response to common lupus autoantigens.

Autoreactivity profiles of PTM-specific histone peptides from the HEMP arrays revealed a transient IgG response in one individual mouse that disappeared by the third month and very little reactivity in the other two mice (Additional file 1: Supplemental Figure 5E). There was little or no reactivity to adjuvanted CRAMP or its human analogue, LL-37. Interestingly, all three mice and the MRL/*lpr *positive control serum exhibited strong IgM and IgG autoreactivity to methyl-H3K18, which decreased somewhat at the three month time point. Methyl-H3K18 is a recently identified methylation mark on histone H3 and has been previously reported to be expressed at reduced levels in MRL/*lpr *splenocytes [51]. Therefore, the targets of autoreactivity observed in SLE and induced by NET immunization overlap partially, with several PTMs distinguishing NET-induced autoreactivity from SLE-autoreactivity profiles.

## Discussion

Since their recent discovery, NETs have been the focus of considerable study examining their roles in innate immunity, and in particular, assessing several putative links to autoimmunity. Histone proteins which are a significant component of NETs, have long been the subject of intense study as they comprise a major class of autoantigens in SLE [52] and are richly decorated with PTMs that dynamically encode epigenetic information in chromatin [53]. However, few studies have characterized the post-translational state of histones within NETs or examined their association with autoimmunity. We tested the hypothesis that NETs and histone PTMs have the capacity to induce autoantibodies that target histones with a focus on SLE.

First, we asked whether histone PTM-specific reactivity could be identified and characterized in SLE, in order to serve as a basis of comparison for any histone PTMs identified in NETs. In comprehensive autoantibody profiling of a well-characterized cohort of patients with SLE within the ABCoN on human epigenome microarrays, we confirmed serum IgG reactivity to acetyl-histone H2B peptides in concordance with previous work [28]. We also observed statistically significant IgM reactivity to multiple H3 and H4 PTM epitopes as well as widespread serum IgM reactivity to methyl-H3 PTM epitopes. One possible explanation is that endogenous histone PTMs may induce a low-level autoantibody response that is present in both healthy and SLE patients and that additional pro-inflammatory signals and T cells help are required to induce autoreactive B cells to affinity maturation and isotype switching to IgG [54]. Surprisingly, serum reactivity to citrullinated epitopes was observed at only low levels for both IgM and IgG. The biological and clinical significance of these findings will require additional and ongoing studies.

To ascertain whether NETs contain SLE serum-reactive PTM antigens, we characterized human and murine-derived NETs using a broad panel of unique, commercially available antibodies recognizing specific histone PTMs. We observed that histones within NETs harbored most of the examined methylation marks, including mono-, di-, and tri-methyl H3 at K4, K9, K27, K36 and H4 at K20. Separately, we identified major trends in the pattern of other histone PTMs enriched in NETs derived from activation of neutrophils using diverse stimuli, including marks associated with transcriptional repression as well as hypercitrullination. This suggests that examining the PTM state of NET chromatin may yield insights into the underlying mechanisms responsible for the profound changes to the chromatin during the process of NETosis.

The hypercitrullination that we observed during NETosis in primary human PMNs and EPRO cells stimulated with diverse stimuli confirms an earlier report of citrullination in HL-60 cell-derived NETs [55]. Consistent with this finding, we observed a corresponding decrease in arginine methylation during NETosis of human and mouse neutrophils, likely reflecting conversion of arginine residues to citrulline. Other, more subtle differences were observed in multiple marks across different conditions; however, given the ECL amplification approach used in most standard immunoblotting approaches, along with the performance of most commercial polyclonal antibodies, it is difficult to ascertain whether such differences are biologically significant.

However, many but not all PTMs that were recognized by serum autoantibodies from SLE patients were found in NETs. Many of the serum-reactive PTMs found in NETs were detected at only modest levels in both SLE histone positive and healthy serum samples, for both IgG and IgM isotypes. Surprisingly, histone PTMs toward which significantly more reactivity was observed in sera from SLE patients known to have anti-histone antibodies (in particular, acetyl-H2B), were absent or detected at only low levels in NETs produced from HL-60 or EPRO cells.

To account for this discordance, one possibility may be that acetylated histones are dissociated during the chromatin condensation step in NETosis-acetylated histones are thought to be in a looser conformation within the nucleosome due to loss of positive charge on acetyl lysine residues [56]. It is also conceivable that these acetyl histones may be particularly immunogenic since they may disperse more widely, increasing their chances of uptake by a professional antigen presenting cell, generating a subsequent proinflammatory response by the adaptive immune system. A second possibility is that only the NETs from a special subpopulation of polymorphonuclear cells are responsible for their immunogenicity in SLE. A recent study described the discovery of low density granulocytes (LDGs) whose greater tendency to undergo NETosis elicited a stronger immune response than conventional neutrophils [57].

Numerous studies have investigated the significance of histone acetylation in SLE, and the pattern of evidence suggests that histone acetylation within cells negatively correlates with disease activity. Splenocytes from MRL/*lpr *mice have hypoacetylated histones H3 and H4 when compared to control MRL/MPJ mice [51] and administration of trichostatin A (a histone deacetylase (HDAC) inhibitor) to MRL/*lpr *mice can be used to improve disease outcome [51,58]. In T cells isolated from SLE patients, global H3 and H4 hypoacetylation was observed when compared to cells obtained from healthy donors [59]. Furthermore, mice with a conditional knock-in of the p300 acetyltransferase gene (in which the histone acetyltransferase activity was absent) develop spontaneous lupus-like disease [60]. Collectively, these studies demonstrate that hypoacetylated histones are associated with increased disease activity and that interventions restoring acetylation in MRL/*lpr *mice improved disease outcome.

Clearly, the acetylation state of histones in disease-relevant cells has a significant bearing on disease outcome; the discordance between observed anti-acetyl histone antibodies in SLE sera and the decrease in acetyl histones in NETs suggest the following three possibilities: (i) NETs are depleted of acetylated histones during NETosis and that these free histones may contribute to the autoimmune response in SLE; (ii) NETs are not the *in vivo *immunogens responsible for the observed patterns in reactivity to histone PTMs; and (iii) NETs derived from LDGs are enriched for acetyl histones and may account for their increased immunogenicity.

In our own mouse studies, we immunized BALB/c mice with NETs or with NETs combined with CRAMP and expected the mice to develop autoantibodies that recognize specific PTMs present in NETs. Here, we tested the hypothesis that NET PTMs are capable of breaking tolerance to self-antigens, including but not limited to histone PTMs, as observed in human SLE. We observed a moderately strong IgM and IgG response to DNA and a modest response to other NET self-antigens, including myeloperoxidase, elastase, and histones. This result is similar to mouse studies performed by Mevorach *et al*. in which mice were immunized with material derived from apoptotic cells, leading to a modest, transient response including the production of antinuclear antibodies, anticardiolipin and anti-dsDNA antibodies, along with increased glomerular IgG deposition [22] and slightly accelerated disease kinetics in MRL/*lpr *and other autoimmune backgrounds [61]. However, while we observed modest but significant and reproducible IgG and IgM reactivity to self-antigens, these mice did not exhibit other features of human SLE nor of autoimmune-prone SLE mouse models. One possible explanation for this observation is that NET chromatin purified *in vitro *is not equivalent to NETs generated *in vivo*, since the latter includes microbial determinants associated with TLR ligands and could thus act as superior adjuvants to break tolerance to self-antigens.

Anti-histone antibodies have also been observed in other diseases. For instance, NETs have been observed in the glomeruli of patients with antineutrophil cytoplasmic antibodies (ANCA) associated vasculitis, perhaps reflecting a common etiology [62]. In drug-induced lupus (DIL), the vast majority of patients exhibit significant positive anti-histone antibody titers primarily targeting histone H2A/H2B [63], concordant with our observed significant reactivity to acetyl and unmodified histone H2B. Extracellular histones can induce septic shock in mice and concurrent infusion of anti-histone H4 antibodies was protective against LPS-induced shock [64]. This finding is consistent with speculation in the field that IgM autoantibodies may play a protective role against an excessive immune response [50]. We evaluated the role of distinctive NET PTMs as a source of self-antigens with a qualitative relationship to autoimmunity. However, a clearance deficiency of NETs has also been described in patients with SLE [4]. Therefore, persistence of NETs, through insufficient clearance by endonucleases or phagocytes, may serve as a complementary quantitative perturbation leading to SLE pathogenesis.

Nucleoprotein complexes are also candidates to be involved in both the etiology and pathogenesis of SLE and murine lupus, wherein they might complement NETs. Many dsDNA autoantibody-producing B cells from SLE patients are thought to be affinity selected by uncleared apoptotic lymphocytes in germinal centers. Therefore, it may not be surprising that NET derived modifications are not the dominant epitopes recognized by anti-histone autoantibodies. Further, in the presence of anti-dsDNA, NETs generated in several tissues may serve as targets for the anti-dsDNA. The binary complex of nucleoprotein and autoantibody may be prone to shift the clearance of dead cell remnants (apoptotic bodies and NET-structures) to inflammation.

Many of the specific post-translational histone modifications enriched in NETs overlapped those to which significant autoreactivity is seen in a subset of patients with SLE. Nonetheless, this overlap was partial, and many PTMs distinguished NETs from SLE-autoreactivity profiles. Further, while NETs were observed to be modestly immunogenic *in vivo*, the induced serological autoimmune responses were distinct from those observed in patients with lupus, as well as autoimmune-prone MRL/*lpr *mice.

## Conclusions

In summary, to investigate the link between NETs and SLE, using histone PTMs as prospective biomarkers, we investigated the serum reactivity profile to a panel of histone PTMs in a cohort of SLE patients and identified significant autoantibody reactivity to acetyl-histone H2B. We devised a methodology to culture myeloid cell lines to produce NETs, and found that their PTMs indicate a state of transcriptionally silent chromatin that has decondensed with the aid of citrullination, a result of the NETosis process. Some of the human SLE serum reactivity overlaps with PTMs found on NETs; however, the presence of autoantibodies against acetyl-histone H2B is discordant with the decrease in histone H2B acetylation in NETs. Furthermore, murine cell line-derived NETs are weak immunogens *in vivo*. This result suggests that the breaking of tolerance to self requires more than a simple exposure to NETs.

## Abbreviations

ABCoN: Autoimmune Biomarkers Collaborative Network; ANCA: antineutrophil cytoplasmic antibody; ATCC: American Type Culture Collection; ATRA: all-trans retinoic acid; CRAMP: cathelicidin-related antimicrobial peptide; DMSO: dimethylsulfoxide; FBS: fetal bovine serum; GM-CSF: granulocyte macrophage colony-stimulating factor; HDAC: histone deacetylase; HEMP: human epigenome microarray platform; HRP: horse radish peroxidase; Ig: immunoglobulin; IL-3: interleukin 3; IMBM: Iscoveís modified dulbeccoís medium; IRB: Institutional Review Board; LDG: low density granulocyte; LPS: lipopolysaccharide; MABA: Multiple Antigen Blot Assay; MFI: mean fluorescence intensity; MNase: micrococcal nuclease; NET: neutrophil extracellular trap; P/S: penicillin/streptomycin; PAD: peptidyl arginine deiminase; PBST: phosphate-buffered saline and tween; pDC: plasmacytoid dendritic cell; PMA: phorbol myristate acetate; PTM: post-translational modification; RA: rheumatoid arthritis; SAM: Significance Analysis of Microarrays; SCF: stem cell factor; SLE: systemic lupus erythematosus; SR: serine/arginine-rich; TBST: tris-buffered saline and tween; TLR9: Toll-like receptor 9; TNF: tumor necrosis factor.

## Competing interests

The authors declare that they have no competing interests related to the publication of this manuscript.

## Authors' contributions

CLL conceived of the study, designed and coordinated its execution, carried out the immunoblot assays, performed statistical analysis for the array studies, and drafted the manuscript. ST carried out array assays, cell culture, NETs visualization, and mouse studies. JR participated in NETs visualization. GM participated in cell culture and immunoblot assays. OG provided the histone peptide libraries used in HEMP and immunoblot resources, including the antibody panels. ECG provided human SLE samples used in this study. AAA helped to design the experiments, to interpret and analyze resulting data and to write the manuscript. PJU participated in the conception of the study, participated in its design and coordination, and helped to draft the manuscript. All authors read and approved the final manuscript.

## Supplementary Material

Additional file 1 **Supplementary Appendix**. Supplementary information containing Supplemental Tables 1-3 and Supplemental Figures 1-5. Click here for file
