# Supplementary material for: Specific post-translational histone modifications of neutrophil extracellular traps as immunogens and potential targets of lupus autoantibodies
Source: Arthritis Res Ther. 2012 Feb 2;14(1):R25. doi: 10.1186/ar3707 (PMC3392818; doi:10.1186/ar3707)
Supplement: Additional file 1 — Supplementary Appendix. Supplementary information containing Supplemental Tables 1-3 and Supplemental Figures 1-5. [file ar3707-S1.PDF]

Supplemental Appendix

This appendix has been provided by the authors to give readers additional information about their work.

Supplement to Liu CL, et al. “Specific post-translational histone modifications of neutrophil extracellular traps as immunogens and potential targets of lupus autoantibodies”

Supplemental Tables & Legends

Supplemental Table 1: NET Yield Estimations

Cell Type	Cell input	Pellet (μg)	NETs (μg)	Ratio	Yield (%)
Primary human neutrophils	4.00E+06	0.08	7.10	0.989	98.9%
HL-60 Low yield	3.00E+06	11.55	1.20	0.094	9.4%
EPRO Low yield	3.00E+06	11.49	2.03	0.150	15.0%
HL-60 High yield	3.00E+06	2.58	2.19	0.459	45.9%
EPRO High yield	3.70E+06	5.93	6.10	0.507	50.7%

DNA yields were determined from the amount of DNA recovered from an aliquot of NETs or remaining pellet following micrococcal nuclease digestion. NET yield was determined to be the ratio of NET DNA recovered over the total DNA recovered from both the NET and pellet fractions. The cell input indicates the starting number of cells used to produce the DNA from the NET and pellet fractions. Data for low and high yielding preparations are shown for HL-60 and EPRO derived NETs. MPRO yield data are not shown since these cells did not efficiently produce NETs.

Supplemental Table 2: Panel of antibodies used in Immunoblot Assay. 41 commercially available antibodies used in the immunoblot assays in **Supplemental Figure 4** and **Figure 4** are summarized here. The dilution factor used for each antibody was empirically determined based on moderate band intensity in undifferentiated HL-60 and EPRO cells (data not shown).

Epitope	Dilution Factor	Ab type	Supplier	Catalog number
H3	1:5000	Rabbit polyclonal	Abcam	Ab1791-100
H4	1:500	Rabbit polyclonal	Abcam	Ab7311-100
H2A	1:200	Rabbit polyclonal	Abcam	Ab18255-100
H2B	1:200	Rabbit polyclonal	Abcam	Ab18977
H3K4Me1	1:1000	Rabbit polyclonal	Abcam	Ab8895-100
H3K4Me2	1:200	Rabbit monoclonal	Abcam	Ab32356-100
H3K4Me3	1:4000	Rabbit polyclonal	Active Motif	39915
H3K9Me1	1:2000	Rabbit polyclonal	Abcam	Ab9045-100
H3K9Me2	1:200	Mouse monoclonal	Abcam	Ab1220-100
H3K9Me3	1:4000	Rabbit polyclonal	Abcam	Ab8898-100
H3K27Me1	1:2000	Rabbit polyclonal	Upstate/Millipore	17-643
H3K27Me2	1:1000	Rabbit polyclonal	Active Motif	39245
H3K27Me3	1:200	Rabbit polyclonal	Upstate/Millipore	07-449
H3K36Me1	1:4000	Rabbit polyclonal	Abcam	Ab9048
H3K36Me2	1:1000	Rabbit polyclonal	Active Motif	39255
H3K36Me3	1:5000	Rabbit polyclonal	Abcam	Ab9050
H3K79Me1	1:1000	Rabbit polyclonal	Abcam	Ab2886-100
H3K79Me2	1:500	Rabbit polyclonal	Abcam	Ab3594-100
H3K79Me3	1:200	Rabbit polyclonal	Abcam	Ab2621-100
H4K20Me1	1:2000	Rabbit polyclonal	Abcam	Ab9051-100
H4K20Me2	1:200	Rabbit polyclonal	Abcam	Ab9052-100
H4K20Me3	1:500	Rabbit polyclonal	Abcam	Ab9053-100
H2BK5Ac	1:1000	Rabbit polyclonal	Abcam	Ab40886
H2BK12Ac	1:200	Rabbit polyclonal	Abcam	Ab40883
H2BK20Ac	1:200	Rabbit polyclonal	Abcam	Ab52988
H3Cit(2,8,17)	1:200	Rabbit polyclonal	Abcam	ab5103
H3Cit26	1:200	Rabbit polyclonal	Abcam	ab19847
H4Cit3	1:500	Rabbit polyclonal	Upstate/Millipore	07-596
H3S10P	1:200	Rabbit polyclonal	Upstate/Millipore	06-570
H3T3P	1:1000	Rabbit polyclonal	Abcam	Ab17352-100
H3K9ac	1:200	Rabbit polyclonal	Upstate/Millipore	06-942
H3K14ac	1:4000	Rabbit polyclonal	Upstate/Millipore	07-353
H3K18ac	1:500	Rabbit polyclonal	Upstate/Millipore	07-354
H3K27ac	1:1000	Rabbit polyclonal	Upstate or Abcam	07-360 or ab61235
H4K5Ac	1:5000	Rabbit polyclonal	Upstate or Abcam	07-327 or ab61236
H4K8Ac	1:4000	Rabbit polyclonal	Abcam	Ab15823-100
H4K12Ac	1:400	Rabbit polyclonal	Upstate or Abcam	07-595 or ab61238
H4K16Ac	1:1000	Rabbit polyclonal	Upstate/Millipore	07-329
H3R2me2a	1:500	Rabbit polyclonal	Abcam	Ab8046-50
H3R17me2a	1:200	Rabbit polyclonal	Abcam	Ab8284-100
H4R3Me2(s)	1:500	Rabbit polyclonal	Abcam	Ab5823

Supplemental Table 3: Proteinuria Measurements

	NETs alone			NETS + CRAMP		
Week	1	2	3	1	2	3
4	+	+	+	+	+	+
6	+	trace	+	+	trace	Trace
7	+	+	trace	trace	+	Trace
8	++	trace	+	trace	+	Trace
9				+	+	Trace
10				+	+	+
11				+	+	Trace
12				+	trace	Trace
13				+	+	+
14				+	+	Trace

Proteinuria measurements were made using dipstick analysis (Albustix; Bayer) on a weekly basis starting at Week 4 for each of 6 mice immunized with NETs alone or with NETS and CRAMP. Proteinuria measurements reflect estimates in mg/dL, with trace = <30, + = 30 – 100; ++ = 100 – 300. Gray cells indicate unavailable data.

Supplemental Figures & Legends

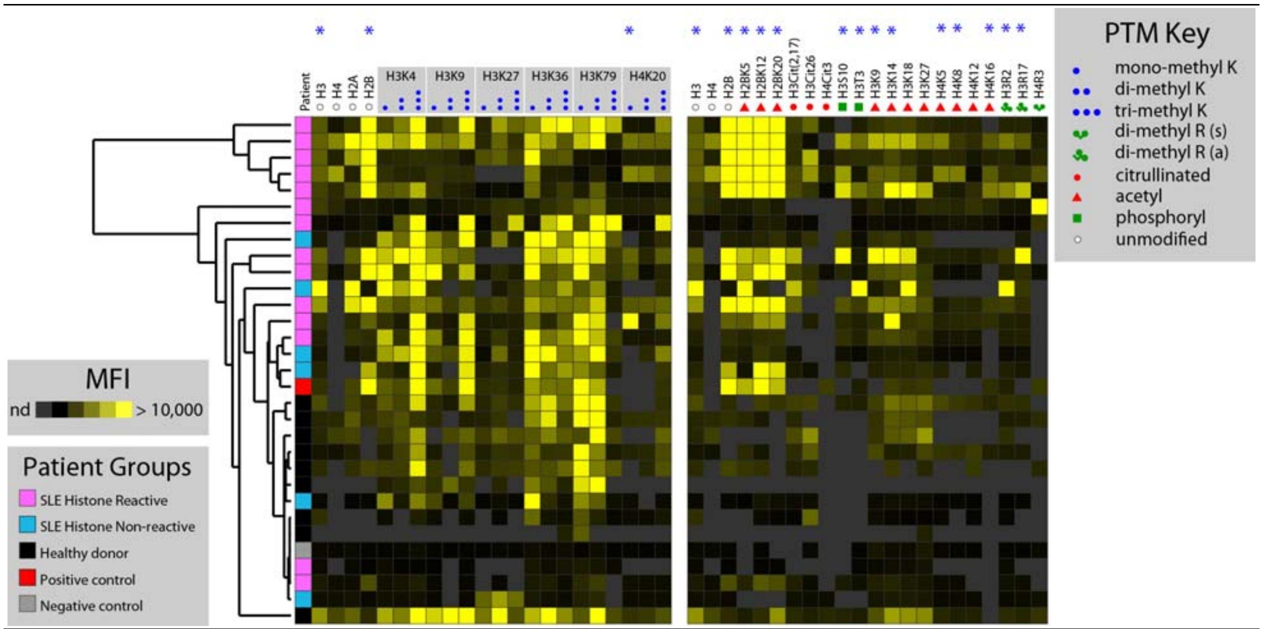

Supplemental Figure 1. Histone-reactive SLE IgM autoantibodies recognize acetylated and methylated histones. Sera from patients with SLE or sera from healthy subjects were profiled on Human Epigenome Microarray Platform (HEMP), and probed with a secondary anti-human IgM antibody. Samples (rows) are clustered hierarchically based on corresponding reactivity profiles to the depicted peptide epitopes from HEMP arrays (columns). The depicted histone peptides (columns) were selected to match those interrogated by immunoblot assays in **Figure 4** and **Supplemental Figure 4**, with specific PTMs epitopes shown at top according to the PTM key. Heatmap tiles reflect magnitude of IgG autoantibody binding reactivity, according to the mean fluorescence intensity (MFI) intensity scale as indicated. The left heatmap panel consists primarily of peptides containing mono-, di- and tri-methyl lysine PTMs, while the right panel consists of peptides containing a variety of PTMs, including citrulline, acetyl, phosphoryl, and both symmetric (s) and asymmetric (a) di-methyl arginine. Blue asterisks (*) mark significant differences autoantibody binding reactivity between histone positive samples and a combined group of histone negative and healthy control samples, as determined by Significance Analysis of Microarrays (SAM). nd, not detected.

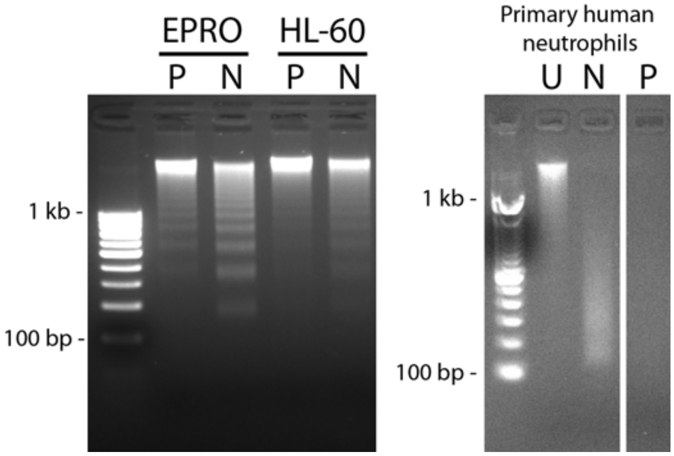

Supplemental Figure 2. Size distribution of DNA from Micrococcal Nuclease Digestion of NETs. NETs (N) or the remaining pellet (P) were loaded onto a 2% agarose gel in Tris-Acetate-EDTA buffer containing ethidium bromide, alongside a 100 bp ladder. For EPRO and HL-60 samples, 250 ng was loaded per lane; for primary human neutrophils, 175 ng was loaded, with DNA isolated from unstimulated neutrophils (U) also included.

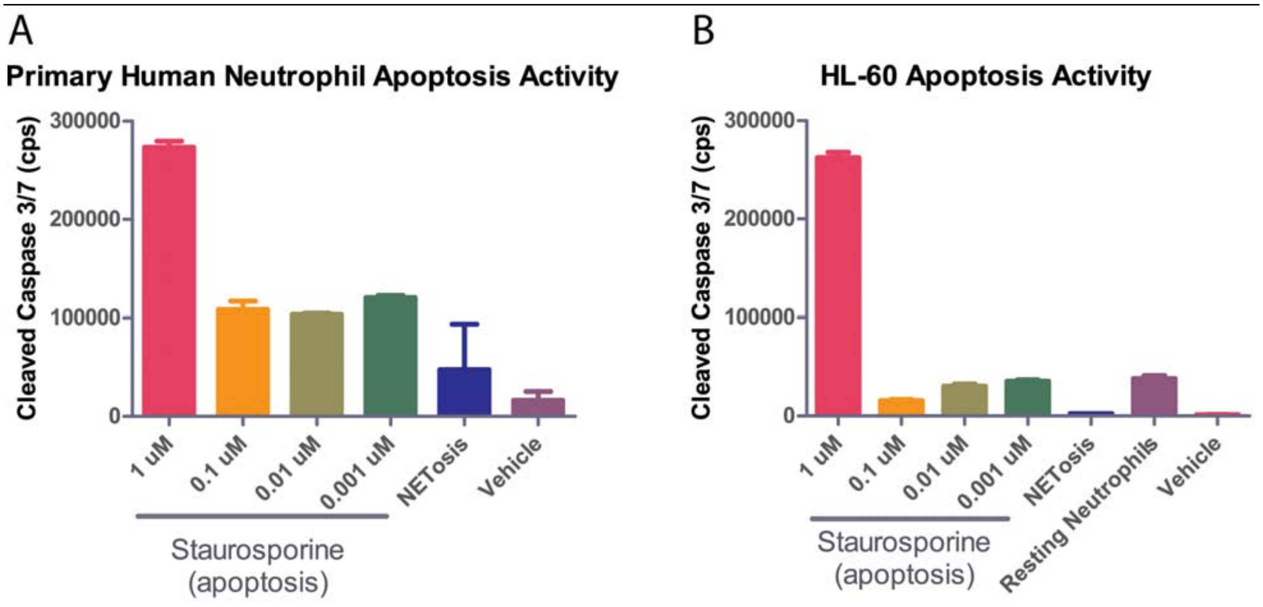

Supplemental Figure 3. Induction of NETosis with hydrogen peroxide does not result in a significant degree of apoptosis. Primary human neutrophils (A) or neutrophils derived from HL-60 cells (B) were plated in triplicate at 2.5 x 105 cells/ml at 100 μl per well in 96-well plates, and induced to undergo NETosis with 10 mM hydrogen peroxide for 4 hours, with staurosporine (0.001 to 1 μM) used as a positive control, and vehicle (cell-free) as a negative control. Apoptosis was measured using a chemiluminescent assay for cleaved caspase 3/7. Results are shown in counts per second (cps). NETosis induced by 4-hour stimulation with hydrogen peroxide stimulation induced significantly less apoptosis than observed in neutrophils “resting” over this period, and also less than the degree of apoptosis induced by staurosporine across the concentration range tested.

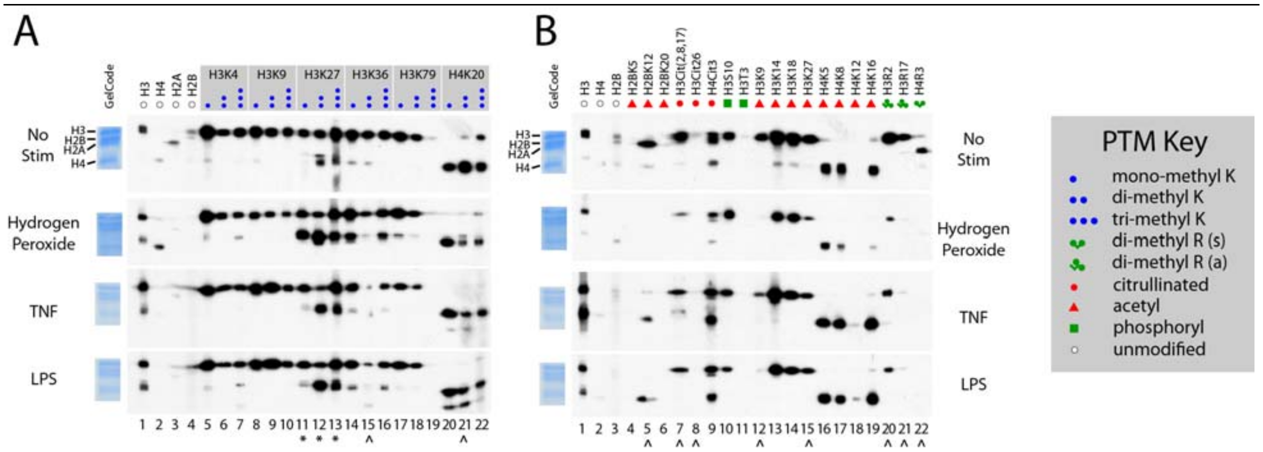

Supplemental Figure 4. HL-60 derived NETs contain histone PTM marks consistent with transcriptional silencing. HL-60 derived neutrophils were stimulated for 4 hours, and NETs were harvested and profiled by MABA, with band migration shown for 10-15 kDa range. A panel of anti-histone antibody epitopes (top) corresponding to the peptides probed in **Figure 1** is shown, with PTMs according to the PTM key. A. Lysine methylation panel. B. Mixed PTM panel, including acetyl, phosphoryl, methyl, and citrulline modifications as depicted in legend. Bottom: asterisks or carats indicate increase or decrease (respectively) of band intensity for indicated lane in NETs compared to unstimulated neutrophils. Film exposure times for all cells were 2’ for no stimulation and stimulation with hydrogen peroxide, and 10’ for TNF or LPS stimulation.

Supplemental Figure 5.

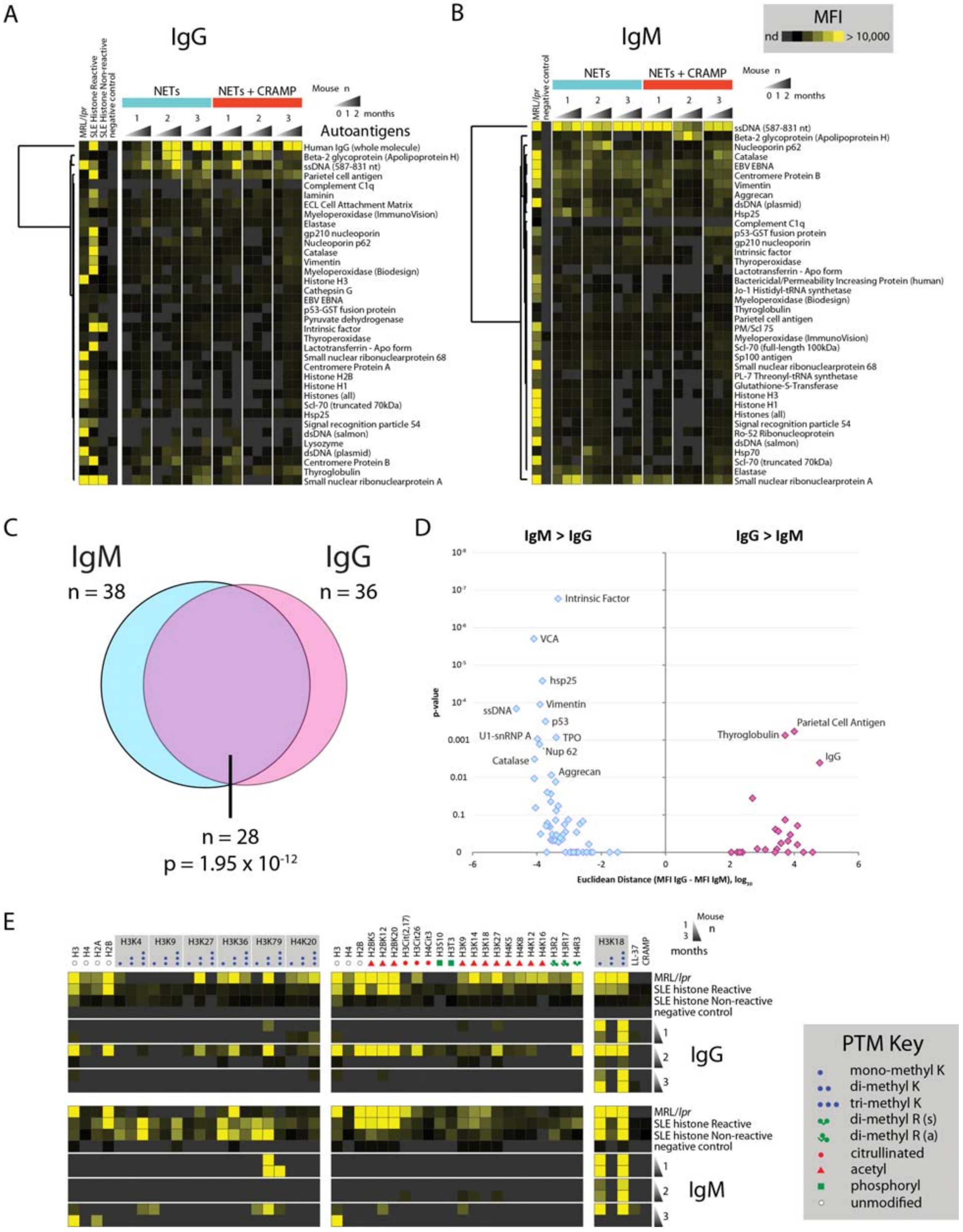

Supplemental Figure 5. Murine NETs induce modest autoantibody responses to diverse antigens. Serum samples from female BALB/c mice, treated with NETs alone or with CRAMP were profiled on autoantigen microarrays probed with anti-mouse IgG (**A**) or IgM (**B**) secondary antibodies. Serum from MRL/*lpr* mice was used as positive control, and omission of primary antibody as a negative control. Mean IgG MFI values from SLE patient groups as defined in **Figure 1** are shown for comparison. Result for auto-antigens (rows) were filtered such that at least two observations had MFI>1000, and then hierarchically clustered. **C**. Significant overlap of autoantigen reactivity between IgG and IgM isotypes, with p-value determined using cumulative hypergeometric distribution. **D**. Significant differential autoantigen reactivity between IgM and IgG isotypes. The Euclidean distance between IgM and IgG profiles was calculated from their MFI values, with nominal p-value determined using a paired two-tailed t test **E.** Serum samples from individual mice treated with NETs in combination with CRAMP were collected at 1 and 3 months and profiled on HEMP microarrays. Individual mice are shown as pairs of rows in chronological order.
